# The effect of sarcopenic obesity and muscle quality on complications after DIEP-flap breast reconstruction

**DOI:** 10.1016/j.heliyon.2022.e09381

**Published:** 2022-05-11

**Authors:** N. Sadok, M.E. Hartmans, G.H. de Bock, J.M. Klaase, P.M.N. Werker, A.R. Viddeleer, L. Jansen

**Affiliations:** a University of Groningen, University Medical Center Groningen, Department of Plastic Surgery, Groningen, the Netherlands; b University of Groningen, University Medical Center Groningen, Department of Surgical Oncology, Groningen, the Netherlands; c University of Groningen, University Medical Center Groningen, Department of Epidemiology, Groningen, the Netherlands; d University of Groningen, University Medical Center Groningen, Department of Hepatobiliary Surgery, Groningen, the Netherlands; e University of Groningen, University Medical Center Groningen, Department of Radiology, Groningen, the Netherlands

**Keywords:** Breast reconstruction, Transplantation, Autologous, Postoperative complications, Risk factors, Muscle, Skeletal/physiopathology, Sarcopenia, Sarcopenic obesity, Body composition, Tomography, X-ray computed

## Abstract

**Introduction:**

The aim of this study was to evaluate whether sarcopenic obesity and muscle quality as expressed by skeletal muscle radiodensity (SMD) are associated to postoperative complications in women undergoing DIEP-flap breast reconstruction (BR).

**Methods:**

All patients who underwent DIEP-flap BR at our tertiary center between 2010 and 2018 were asked to sign informed consent for the use of their electronic medical records and images. By outlining anatomical skeletal muscle contours on the preoperative abdominal CT-scan at lumbar level L3, SMD and skeletal muscle indices (SMI) were measured by two observers independently. Using logistic regression analyses, the association between sarcopenic obesity (BMI >25 & SMI <39), low SMD (<40HU), and Clavien-Dindo (CD) grade ≥ II complications was evaluated. In this way odds ratios (OR) and adjusted odds ratios (OR_adjusted_) were provided.

**Results:**

Out of the 103 patients included in this study, 36% had CD grade ≥ II complications within 30 days of surgery. Twenty patients (19%) suffered from sarcopenic obesity of whom eleven patients (55%) had CD grade ≥ II complications (OR = 2.7, p = 0.05). In a multivariate analysis, sarcopenic obesity was not significantly related to a higher complication rate (OR_adjusted_ = 2.2, p = 0.14) but women with SMD below average and those with prior radiotherapy had a higher risk for grade ≥ II complications (OR_adjusted_ = 2.9, *p = 0.02* and OR_adjusted_ = 2.7, *p = 0.02* respectively).

**Conclusion:**

Below average SMD (<40HU) was found to be associated with the development of postoperative CD grade ≥ II complications in women undergoing DIEP-flap BR. Future research should evaluate whether improving SMD reduces the complication incidence in this patient group.

## 1 Introduction

Autologous breast reconstruction (BR) has gained popularity worldwide. It is associated with higher patient satisfaction and quality of life compared to alloplastic BR with implants [1]. One of the most commonly applied autologous BR techniques is the Deep Inferior Epigastric Perforator flap (DIEP) [2]. Although the DIEP-flap is superior to alloplastic BR when it comes to patient satisfaction and overall QoL [3], it implies major surgery which bears a higher risk of complications [4]. Women qualifying for this type of BR need to have sufficient abdominal subcutaneous fat surplus to reconstruct the new breast(s). Consequently, these women usually have a higher Body Mass Index (BMI) than considered to be optimal for physical health (BMI 20 to 25) [5]. Multiple studies have confirmed that higher BMI increases the risk of postoperative complications. This results in a paradox when selecting the optimal reconstruction technique for women who want to undergo BR [6, 7, 8, 9, 10]. However, not all women with higher BMI develop postoperative complications and other health parameters may play a role in this matter.

Sarcopenic obesity as such may be a relevant health parameter in this patient group. It is defined as sarcopenia in the obese, indicating high fat tissue mass with low lean body mass [11, 12]. Research has shown that sarcopenic obesity and reduced muscle quality as expressed by decreased skeletal muscle radiodensity (SMD), or radiation attenuation, have negative effects on the postoperative course after major surgery, and are related to a higher risk of developing complications and poorer survival [11, 12, 13, 14, 15, 16, 17, 18, 19, 20, 21, 22]. The effects of these parameters were mainly investigated in elderly and chronically ill patients. However, measuring sarcopenic obesity and muscle quality may also help distinguish the healthy obese from those who indeed have an increased risk of complications within younger patient groups.

The aim of this study was to assess whether sarcopenic obesity and muscle quality as expressed by SMD are associated with postoperative complications that lead to medical or surgical intervention in the relatively young and healthy women undergoing DIEP-flap BR.

## 2 Material & methods

### 2.1 Context

This retrospective cohort study was executed at a tertiary referral center by researchers from the department of plastic surgery and surgical oncology in collaboration with the departments of radiology and epidemiology. Ethical approval for this study was obtained from the Medical Ethics Review Committee (METc 2018/666). The METc stated that the National Medical Research Involving Human Subject Act (WMO) does not apply to this study.

### 2.2 Study population, inclusion & exclusion

All patients who underwent a unilateral or bilateral DIEP-flap BR in the period between 2010 and 2018 were asked to sign informed consent for the use of their medical records for this study. Exclusion criteria for participation were: no informed consent, the absence of a standardized pre-operative CT-scan and missing data concerning body length needed to interpret the CT-values.

### 2.3 Data collection

Patient characteristics, CT-scans and data on complications were retrieved from the electronic medical record system. The CT-scans were acquired on a Siemens SOMATOM Definition (AS, Edge, Flash), Force, or Sensation (Siemens Medical, Erlangen, Germany) according to this standardized preoperative DIEP-flap protocol: following intravenous contrast administration, the target area was scanned in the arterial phase, with slice thickness of 5 ​mm, and a 512 × 512 matrix. The CT-scan images were anonymized and stored in 16-bit DICOM format for further processing.

For measuring the CT-based body composition parameters, in-house developed software (SarcoMeas 0.46) was used. Three skeletal muscle groups i.e.: the abdominal wall and the two psoas muscles, were manually outlined. Within these drawn outlines skeletal muscle radiodensity (SMD) and skeletal muscle indices (SMI) were calculated. Muscle tissue was defined using the standard Hounsfield Units (HU) ranges for muscle (HU range of -29 to 150), according to international radiological criteria [23]. The SMI is an estimate of muscle volume related to body length and is calculated by dividing the muscle surface areas on CT in cm^2^ by the squared patient length in meters, resulting in an SMI (for abdominal wall and the two psoas muscles together) expressed in cm^2/^m^2^.

**Figure 1:**
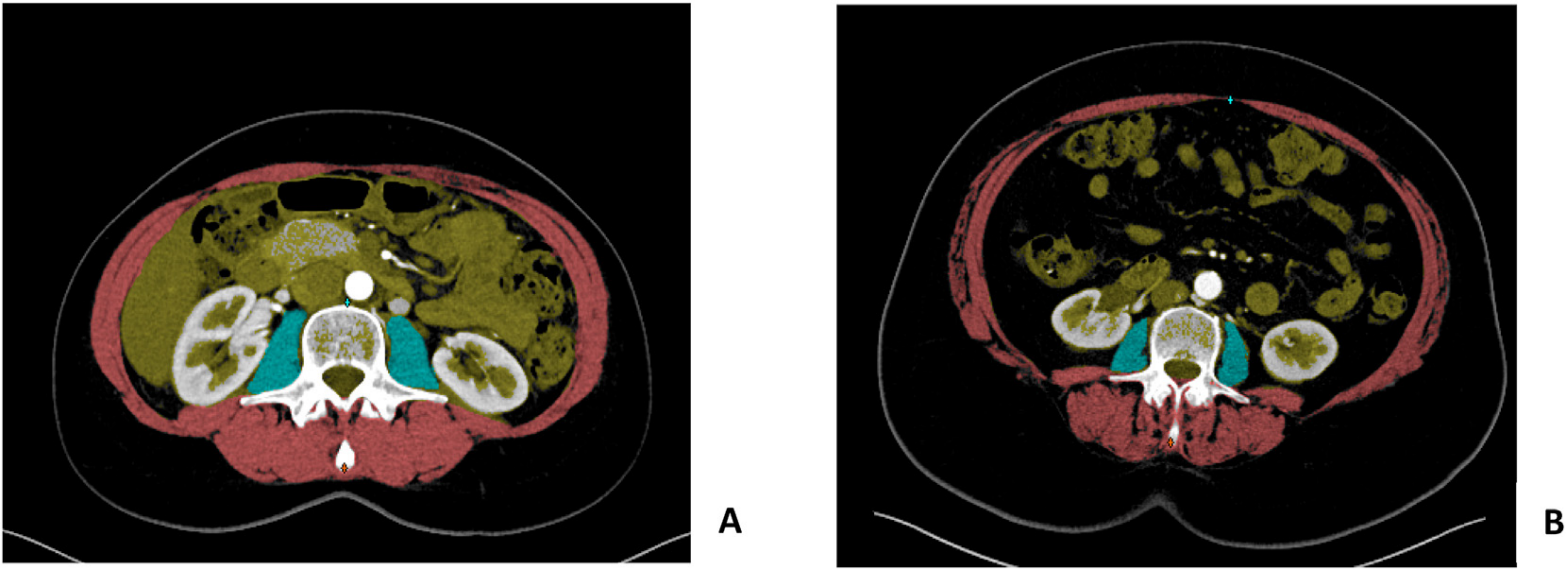
Anatomical outlines acquired with SarcoMeas of the preoperative thoracoabdominal CT-image at lumbar level L3, transverse section. These images show the method of measuring skeletal muscle density (SMD) and SMI and are an example of the anatomical variation related to muscle and fat ratio's between two women undergoing breast reconstruction. The left images (A) show an SMD above the mean (48HU), the image on the right (B) shows a low SMD (26HU). Blue areas: left and right psoas muscle. Red areas: abdominal wall, skeletal muscles. Yellow areas: intra-abdominal organs and fat.

Higher SMD and SMI values indicate higher muscle mass (kg), where lower SMD and SMI indicate fatty infiltration and muscle wasting respectively (Figure 1). The SMD in HU was calculated of the total measured skeletal muscle area. A SMD value below 30HU is considered abnormal/unhealthy [24]. Sarcopenic obesity was defined as BMI>25 & SMI <39 based on the literature [11, 12]. All measurements were acquired using the cross-sectional CT-slice at the lumbar level L3 in which both transverse processes were visible [23, 24], and were executed by two observers independently (N. S. and M.E.H.) to evaluate the inter-observer agreement.

### 2.4 Outcomes

The primary outcome measure was the occurrence of complications within 30 days of the DIEP-flap surgery that needed a medical or surgical intervention. All complications were classified according to Clavien-Dindo (CD) which grades complications related to surgery from I (self-limiting) to V (death) [25]. Complications varied from wound complications to systemic complications. Local complications were registered for both the reconstructed breast(-s) and the abdominal donor site separately. These were complications such as hematoma, seroma, infection and (partial) necrosis. In patients undergoing bilateral BR a computerized randomization tool in SPSS version 26.0 (IBM, NY, USA) was used to select one breast per patient to avoid bias in calculating the complication rate and risk factors for bilateral procedures compared to unilateral surgery. In case of multiple complications, the most severe event was used. The self-limiting CD grade I complications were excluded from the analyzes because of our focus on complications that led to medical or surgical intervention.

### 2.5 Determinants

The following determinants were analyzed in relation to complications that lead to medical or surgical intervention (CD grade ≥ II) complications: age, sarcopenic obesity, SMD, reconstruction indication, reconstruction timing and radiotherapy. Age was calculated in years on the day of the reconstruction. Sarcopenic obesity was categorized into yes or no. SMD was categorized in below or above the mean, as suggested in literature [24]. Reconstruction indication was categorized into BR following prophylactic or therapeutic mastectomy. Reconstruction timing was categorized in BR surgery in the same operation as mastectomy (immediate) and BR at a later stage (delayed). Prior radiotherapy was scored as previous treatment if radiotherapy was applied previously to the recipient site of the DIEP-flap.

### 2.6 Power analysis

For the main outcome model, six parameters (age, sarcopenic obesity, SMD, reconstruction indication, reconstruction timing and prior radiotherapy) were evaluated for their potential effect on the primary outcome. To determine the sample size the rule of thumb was used, whereby ten events per predictor are needed to generate sufficient statistical power and avoid high variability [26]. Assuming that the CD grade ≥ II complication rate would be 50% [4], a sample size of ∼120 patients was needed.

### 2.7 Statistical analysis

Baseline characteristics and data on complications were described using descriptive statistics by means and standard deviations (SD) for normally distributed continuous variables. For non-normally distributed continuous variables and ordinal variables, medians and interquartile ranges (IQR) were used. For the inter-observer agreeability of the measurements, a reliability analysis was used by calculating the Intraclass Correlation Coefficient (ICC) using an ANOVA model. Univariate and multivariate logistic regression analyzes, using backward elimination were applied to estimate odds ratios (ORs) and 95% confidence intervals (CIs) to analyze the association between possible risk factors such as sarcopenic obesity and CD grade ≥ II complications. We performed a sensitivity analysis in which we used a different definition for sarcopenic obesity also used in the literature in which sarcopenic obesity was defined as Visceral abdominal fat (VAT) > 140 cm2 & SMI <39 [27]. P-values of ≤0.05 were considered statistically significant. All statistical analyzes were performed using SPSS version 26.0 (IBM, NY, USA).

## 3 Results

### 3.1 Study population

**Figure 2:**
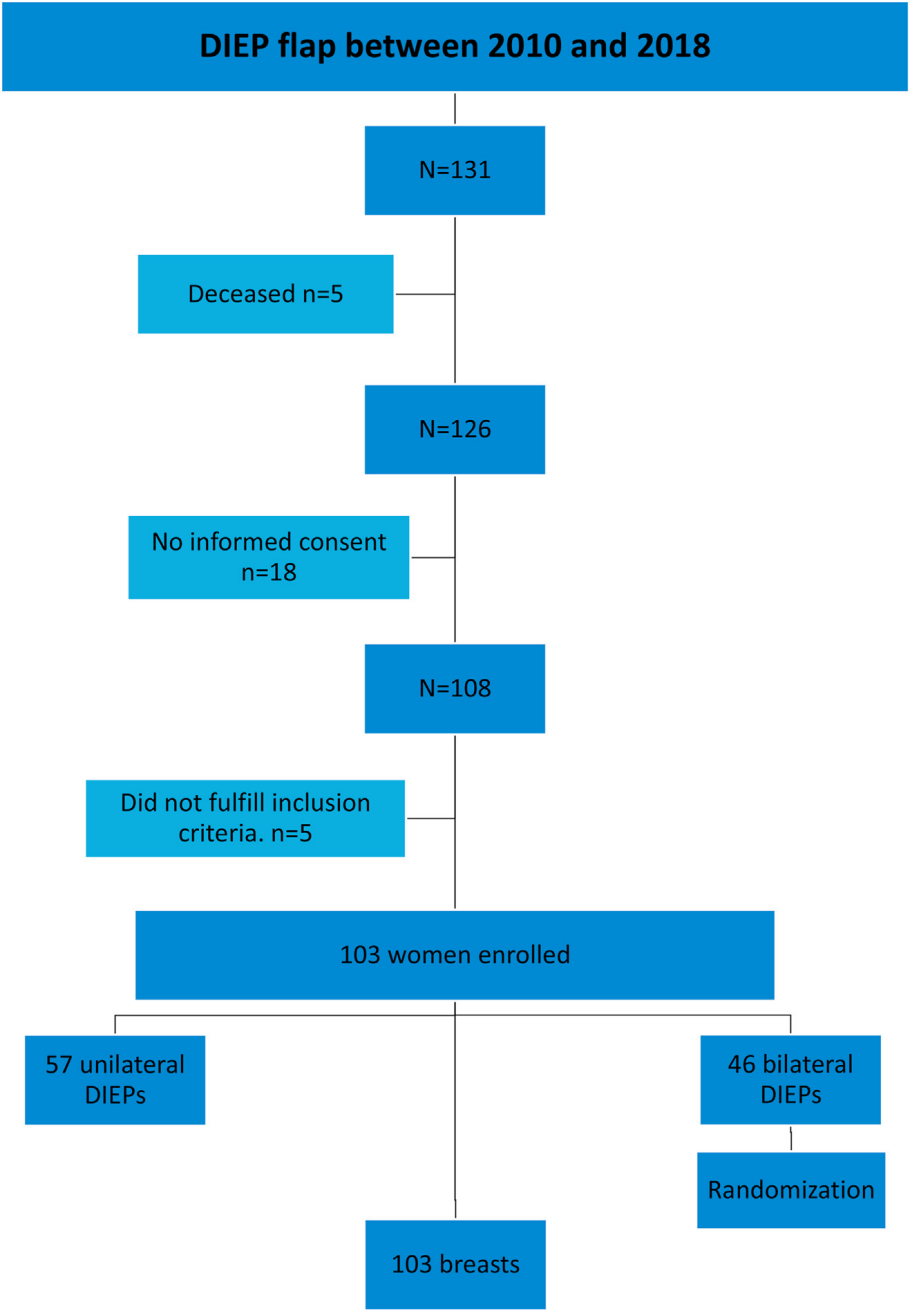
Flowchart of patient inclusion and randomization in women undergoing bilateral reconstruction included in this study.

In total, 131 patients underwent DIEP-flap BR in the period between 2010 and 2018. Five were deceased during follow-up due to recurrent breast cancer, resulting in 126 patients eligible for inclusion. Of the 126 patients, 108 patients (86%) gave informed consent for the participation in this study. After screening patients for exclusion criteria, 103 patients were enrolled (103/131 = 79%). Almost half of women (46/103 = 45%) underwent bilateral reconstruction. See Figure 2 for a flowchart of the patient inclusion. After randomization, in total 103 breasts were included of which thirty breasts (29%) were reconstructed immediately following mastectomy and 73 delayed (71%). Participants were aged between 28 and 67 with a median of 48 (IQR 41–55). The median BMI of the women at reconstruction was 27 (IQR 25–30). The mean time between the CT-scan and reconstructive surgery was 41.5 weeks with a minimum of 1 and a maximum of 124 weeks. The CT-based body composition parameters were normally distributed; the mean SMD value was 40HU and the mean total SMI was 41 cm^2^/m^2^. Twenty patients (19%) met the criteria of sarcopenic obesity as BMI>25 & SMI <39. Eight patients (8%) met the alternative criteria of sarcopenic obesity as VAT>140 cm2 & SMI <39. The number of patients who showed SMD below the aforementioned healthy value of 30HU [24], was 11 (11%). Thirty-four (33%) patients had SMI below healthy values (SMI<39 cm^2^/m^2^). See Table 1 for all patient characteristics.

**Table 1:** Patient characteristics.

Characteristics	Total n = 103
Age, in years, mean (SD)	47.8 (9.3)
BMI at CT-scan, in kg/m^2^, mean (SD)	27.2 (3.1)
BMI at surgery, in kg/m^2^, mean (SD)	27.7 (3.1)
Smoking at time of surgery, n (%)	6 (6)
**Unilateral reconstruction, n (%):**	**57 (55)**

Indication for mastectomy	
- Prophylactic	3 (5)
- Therapeutic∗	54 (95)
Timing of reconstruction	
- Immediate reconstruction	5 (9)
- Delayed reconstruction	52 (91)
**Bilateral reconstruction, n (%):**	**46 (45)**

Indication for mastectomy	
- Bilateral prophylactic	21 (46)
- Bilateral therapeutic	7 (15)
- Unilateral therapeutic and contralateral prophylactic	18 (39)
Timing of reconstruction	23 (50)
- Bilateral immediate reconstruction	
- Bilateral delayed reconstruction	12 (26)
- Unilateral immediate with unilateral delayed reconstruction	11 (24)

Relevant medication×, n (%)	20 (19)
- Acetylsalicylic acid/anticoagulants	1
- Corticosteroids/other anti-inflammatory drugs	6
- Thyroid supplements	7
- Anti-hypertensive drugs	8
- Anti diabetics	1

Prior treatment	
-Chemotherapy,n (%)	45 (44)
-Hormonal therapy,n (%)	52 (51)
-Radiotherapy#^,^ n (%)	41 (40)

Skeletal Muscle Index: in cm2/m2, mean (SD):	41.3 (4.5)
Total Skeletal Muscle Radiodensity: in HU, mean (SD)	39.9 (7.5)
Sarcopenic obesity (BMI>25 & SMI<39)4, n (%)	20 (19.4)

n = number, % = percentage, SD = standard deviation, CT = computed tomography.

∗ = Mastectomy for breast cancer and/or ductal carcinoma in situ (DCIS).

× = The number of patients with relevant medication listed exceeds the total number of patients which had such medication since some patients had a combination of medication.

# = no radiotherapy after reconstruction. In all but one patient at least 12 months passed by between the radiation therapy and reconstruction. The mean time between radiation therapy and reconstruction was 35 months.

### 3.2 Complications

Of the 103 patients included in this study, 81 (81/103 = 79%) had a complication within 30 days of the DIEP surgery. Thirty-seven (36%) had a CD grade ≥ II complication, meaning that 46%(37/81) of patients who had a complication needed additional treatment. In total, 28 patients (27%) had CD grade III-IVa complication. None of the patients had a higher grade complication. Most complications where wound complications (30/36 = 83%) except for two cases of pulmonary embolism, three cases of thrombosis in the vascular anastomosis of the flap (which led in two cases to flap loss) and one case of renal failure. See Table 2 and Table 3 for the specific complications in the study population.

**Table 2:** Type of complications.

Complication Type	Below average SMI N = 50 (49%) N (%)	Above average SMI N = 53 (51%) N (%)	p-value∗	Sarcopenic Obesity N = 20 (19%) N (%)	No Sarcopenic Obesity N = 83 (81%) N (%)	p-value	Below average SMD N = 49 (48%) N (%)	Above average SMD N = 54 (52%) N (%)	p-value
None	18 (36)	23 (45)	0.286	5 (20)	36 (43)	0.104	18 (37)	23 (43)	0.114
Wound dehiscence	6 (12)	2 (4)	0.153	3 (15)	5 (6)	0.183	5 (10)	3 (6)	0.718
Hematoma	9 (18)	9 (17)	0.548	1 (5)	17 (20)	0.186	9 (18)	9 (17)	0.512
Seroma	5 (10)	6 (11)	0.541	3 (15)	8 (10)	0.443	7 (14)	4 (7)	0.531
Necrosis	4 (8)	4 (8)	1.00	2 (10)	6 (7)	0.651	4 (8)	4 (7)	1.00
Infection	4 (8)	3 (6)	0.710	3 (15)	4 (5)	0.131	5 (10)	2 (4)	0.441
Pulmonary Embolism	2 (4)	-	0.233	1 (5)	1 (1)	0.352	1 (2)	1 (2)	1.00
Cellulitis	-	2 (4)	0.496	-	2 (2.5)	1.00	2 (4)	-	0.496
Bleeding	-	2 (4)	0.496	-	2 (2.5)	1.00	-	2 (4)	0.224
Kidney	-	(2)	1.00	-	1 (1)	1.00	-	1 (2)	0.476
Anastomotic failure	2 (4)	1 (2)	0.610	2 (10)	1 (1)	0.096	3 (6)	-	0.244

∗Chi-squared test. Sarcopenic obesity defined as BMI>25 & Skeletal Muscle Index <39.0.

SMI = Skeletal muscle index/muscle volume below and above 41.3 (mean = 41.3 and median = 41.5) SMD = skeletal muscle density/radiation attenuation below and above 40 HU; (mean = 39.9 HU and median = 40.1 HU).

**Table 3:** Univariate and multivariate logistic regression analysis on Clavien Dindo ≥ II complications.

Characteristics	Univariate		Multivariate		Final model with backward selection	
	OR (95% CI)	P-value	OR (95% CI)	P-value	OR (95% CI)	P-value
Age	1.0 (1.0; 1.1)	0.24	1.0 (0.9; 1.1)	0.86	-	-
Sarcopenic Obesity (BMI)	2.7 (0.9; 7.3)	***0.05***	2.4 (0.8; 7.3)	0.11	2.2 (0.8; 6.5)	0.14
SMD <40HU	2.8 (1.2; 6.6)	**0.01**	3.1 (1.2; 8.0)	**0.02**	2.9 (1.2; 7.0)	**0.02**
Prophylactic reconstruction	0.8 (0.3; 1.9)	0.61	1.3 (0.2; 9.4)	0.77	-	-
Immediate reconstruction	0.9 (0.3; 2.1)	0.73	1.0 (0.2; 8.1)	0.97	-	-
Prior radiotherapy	2.5 (1.1; 5.8)	**0.03**	3.1 (1.1; 8.6)	**0.02**	2.8 (1.2; 6.7)	**0.02**

Sarcopenic obesity defined as BMI>25 & Skeletal Muscle Index <39.0.

SMI = Skeletal muscle index/muscle volume below and above 41.3 (mean = 41.3 and median = 41.5) SMD = skeletal muscle density/radiation attenuation below and above 40 HU; (mean = 39.9 HU and median = 40.1 HU) Prophylactic reconstruction (=1) versus therapeutic reconstruction (=0). Immediate reconstruction (=1) versus delayed reconstruction (=0). Relevant medication = anti-inflammatory drugs, thyroid supplements, anti-coagulants, anti-hypertensive drugs, anti-diabetics. Radiotherapy = radiation therapy in medical history of patient at chest area.

### 3.3 Inter-observer correlation

Overall, an excellent (0.954) inter-observer agreement was found in the SMD and SMI measured values.

### 3.4 Factors associated with the occurrence of complications

**Figure 3:**
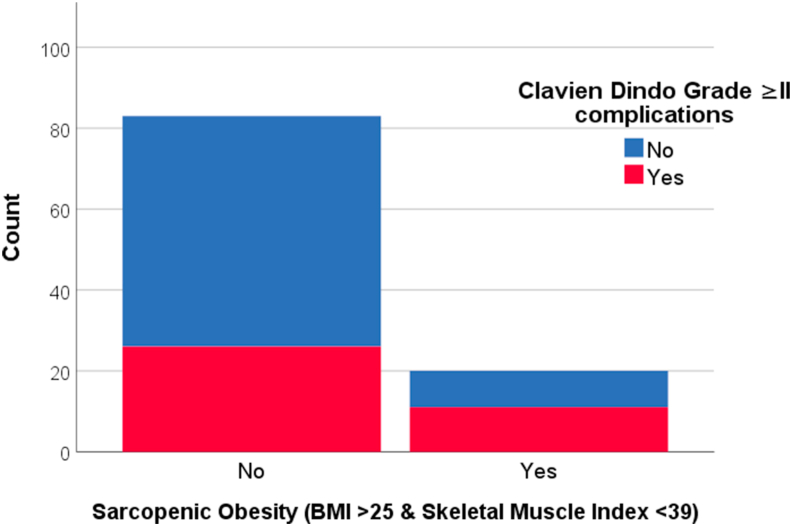
Complication incidence, defined as Clavien-Dindo Grade II and higher, in women without Sarcopenic Obesity (body mass index ≤25 & Skeletal Muscle Index < / ≥39 cm^2/^m^2^) and in women with Sarcopenic Obesity (body mass index >25 & skeletal muscle index <39 cm^2/^m^2^).

**Figure 4:**
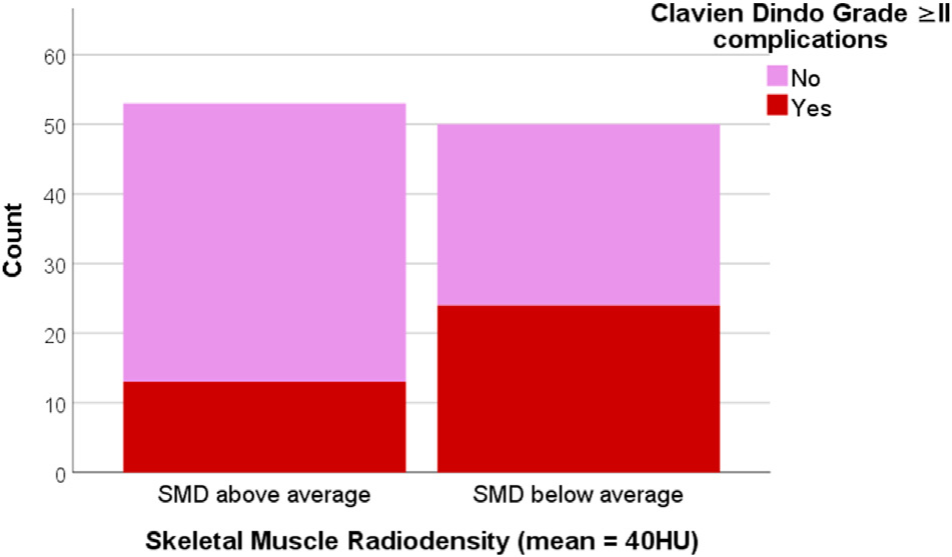
Complication incidence, defined as Clavien-Dindo Grade II and higher, in women with skeletal muscle radiodensity above mean (>40 Hounsfield Units) and women with skeletal muscle radiodensity below mean (<40 Hounsfield Units).

Women with sarcopenic obesity (as BMI>25 & SMI <39) more often had CD grade ≥ II complications compared to women without sarcopenic obesity (55% versus 31%, OR = 2.7, p = 0.05) (Table 4 & Figure 3). Women with SMD values below average (<40HU) had a higher chance of having complications CD grade ≥ II (48% versus 25%, OR = 2.8, p = 0.01) (Table 4 & Figure 4). In multivariate regression analysis, women with SMD below average and women who received radiotherapy had a higher chance for complications CD grade ≥ II (OR_adjusted_ = 2.9, 95% CI 1.2 to 7.0, *p = 0.02* and OR_adjusted_ = 2.8, 95% CI 1.2 to 6.7, *p = 0.02* respectively). Sensitivity analysis for sarcopenic obesity as VAT>140 cm2 & SMI <39 showed similar results (data not shown).

**Table 4:** Univariate and multivariate logistic regression analysis on Clavien Dindo ≥ II complications.

Characteristics	Univariate		Multivariate		Final model with backward selection	
	OR (95% CI)	P-value	OR (95% CI)	P-value	OR (95% CI)	P-value
Age	1.0 (1.0; 1.1)	0.24	1.0 (0.9; 1.1)	0.89	-	-
Sarcopenic Obesity (VAT)	6.1 (1.2; 32.5)	**0.03**	5.6 (0.9; 34.6)	0.06	4.9 (0.9; 28.1)	0.07
SMD <40HU	2.8 (1.2; 6.6)	**0.01**	3.1 (1.2; 8.1)	**0.02**	2.9 (1.2; 7.0)	**0.02**
Prophylactic reconstruction	0.8 (0.3; 1.9)	0.61	1.3 (0.2; 9.4)	0.77	-	-
Immediate reconstruction	0.9 (0.3; 2.1)	0.73	1.0 (0.2; 8.1)	0.97	-	-
Prior radiotherapy	2.5 (1.1; 5.8)	**0.03**	3.1 (1.1; 8.6)	**0.02**	2.8 (1.2; 6.7)	**0.02**

Sarcopenic obesity defined as Visceral Adipose Tissue (VAT) ≥140 cm2 & Skeletal Muscle Index <39.0.

SMI = Skeletal muscle index/muscle volume below and above 41.3 (mean = 41.3 and median = 41.5) SMD = skeletal muscle density/radiation attenuation below and above 40 HU; (mean = 39.9 HU and median = 40.1 HU) Prophylactic reconstruction (=1) versus therapeutic reconstruction (=0). Immediate reconstruction (=1) versus delayed reconstruction (=0). Relevant medication = anti-inflammatory drugs, thyroid supplements, anti-coagulants, anti-hypertensive drugs, anti-diabetics. Radiotherapy = radiation therapy in medical history of patient at chest area.

## 4 Discussion

This study shows that SMD and radiotherapy are independently associated with CD grade ≥ II complications after DIEP-flap surgery (OR_adjusted_ = 2.9, *p = 0.02* and OR_adjusted_ = 2.8, *p = 0.02* respectively). Women with sarcopenic obesity (BMI>25 & SMI <39, n = 20 (19%)) more often had CD grade ≥ II complications (*55% versus 31%*). In multivariate logistic regression analysis, adjusting for SMD and radiotherapy, sarcopenic obesity was not statistically significantly related to CD grade ≥ II complications (OR_adjusted_ = 2.2, p = 0.14). The results were equal regardless which definition of sarcopenic obesity was used. Although, in the sensitivity analysis using visceral abdominal tissue instead of BMI (VAT>140 cm2 & SMI <39, n = 8 (8%)) we found a stronger correlation with the complication rate (OR_adjusted_ = 4.9, p = 0.07). It is possible that we did not find a statistically significant correlation in multivariate regression analysis due to the relatively small sample size. Besides, most women suffering from sarcopenic obesity also have lower SMD [28].

This is the first study assessing sarcopenic obesity (BMI ≥25 & SMI<39) and SMD in women undergoing DIEP-flap BR and one of the very few looking at body composition parameters in women undergoing BR. There is some research on the effect of sarcopenia defined as SMI<38.5, and complications after DIEP-flap BR which found contradictory results [29, 30]. In one of these studies an increased complication incidence, hospital stay and ICU length of stay was found in women with sarcopenia versus women without sarcopenia [29]. In the other study, no difference in complication incidence was found related to sarcopenia [30]. The relation between sarcopenic obesity, decreased SMD and increased risk of postoperative complications has been recognized earlier among patients undergoing other types of major surgery. A study on morbidity after rectal cancer surgery found similar results as our study with higher incidence of complications CD grade ≥ III among patients with sarcopenic obesity (OR_adjusted_ = 3.77, 95%CI = 1.1; 12.7) [11]. Some studies on SMD used different absolute cut-off values for SMD and are thereby difficult to compare to our results [29, 30, 31, 32]. In one study assessing SMD in patients treated for rectal cancer, outcome was also compared for those with SMD below and above the median, similar to the current study, which was 40HU for our study group. They also found that a lower radiation attenuation (SMD) was independently associated with overall (*p = 0.003*) and CD grade ≥ III complications (p = 0.002) [11]. These findings are now confirmed in our population with relatively healthy patients undergoing breast reconstruction.

Another point of argument is that it is possible that there are differences in outcome with differences in body composition amongst different ethnic groups. This indeed confirms the need for a clinically more specific and relevant measure for sarcopenia such as SMD instead of BMI. What would be in line with our findings would be that the general outcome in different ethnic groups would be different if the general body composition is different.

Besides SMD, we confirmed that prior radiotherapy increases the risk of complications, which is comparable to what is described in the literature [4, 6, 7, 8, 9]. This may be due to negative effects of radiotherapy on the microvasculature and wound healing.

### 4.1 Strengths and limitations

One of the main strengths of this study is the limited chance of inclusion bias. Almost 80% of women that underwent a DIEP-flap BR at the study center were included in this study. We expect that our complication registration is relatively complete [33], as patients are being monitored intensively after this type of BR with the main focus on wound healing and as a result, more than half of the registered complications were grade I complications. Another strength is the use of randomization to select only one breast per patient to avoid bias in the calculation of the complication rate and risk factors for bilateral procedures compared to unilateral surgery. In other research it is often unclear how researchers dealt with the analysis of complications in bilateral surgery [29, 30]. More appropriate statistical analysis would have been multilevel analysis, but unfortunately the study group was too small to perform such complex statistical analysis. Furthermore, by eliminating one breast in bilateral surgery, the disadvantage was that the number of complications per patient could not be analyzed. Randomization was preferred because of the advantage that the effect of risk factors that applied only to one of the breasts like reconstruction indication, technique and radiotherapy, could be properly included in the analysis.

A strength of the study was the high quality of the CT-scan measurements. All CT's were scanned according to the same protocol and the inter-observer agreement for the measured values of SMD and SMI was excellent (0.954) [34]. All measurements were performed at the level of the third lumbar vertebra, which has been found to be the preferred level for these measurements [35]. It has been recommended to use CT-scans scanned in the porto-venous phase [35]. The CT's used in the current study were all scanned in the arterial phase. Previous research showed however no significant difference in SMD between the arterial and porto-venous phases [35]. Furthermore, as in this study the mean SMD was used as cut-off point for the analyzes, this choice can be expected to be of no consequence for the study outcome.

### 4.2 Recommendations

Sarcopenic obesity and SMD could be of value when weighing the surgical risks against the benefits. Larger studies, preferably multi-center studies, are needed to further assess the effect of sarcopenic obesity in women undergoing DIEP-flap BR. Besides the effect on the complication rate, SMD was also found to be a prognostic factor for overall survival in breast cancer patients in other research [36]. This might suggest that breast cancer patients with a high SMD before DIEP-flap BR would possibly both have a less complicated postoperative course and may even have a better overall survival [36]. This makes them excellent candidates for DIEP-flap BR.

The question rises whether improving the muscle density by exercising could improve surgical outcome. This idea fits in the current era, where healthcare providers are looking into the development of prehabilitation programs in order to improve patients physical fitness before surgery [37]. To measure physical fitness more accurately, the anaerobic threshold could be determined with a cardiopulmonary exercise test (CPET), but this test is not commonly performed. Future research should evaluate whether improving the physical fitness indeed improves SMD and thereby the surgical outcome of women undergoing DIEP-flap BR. Since CT-scans expose patients to radiation, other tools to assess sarcopenic obesity, as for example hand grip strength and bioelectrical impedance analysis, could possibly aid in assessing progress after intervention for improved physical fitness has been initiated [38, 39].

## 5 Conclusion

In this study, in multivariate analyses, low SMD (<40HU) and prior radiotherapy, were found to increase the risk of CD grade ≥ II complications. Besides, women with sarcopenic obesity seemed to have CD grade ≥ II complications more often compared to women without sarcopenic obesity (55% vs. 31%, p = 0.05, univariate analysis). Future research should evaluate whether improving SMD could reduce the complication incidence in this patient group.

## Declarations

### Author contribution statement

Nadia Sadok and Alain R Viddeleer: Conceived and designed the experiments; Performed the experiments; Analyzed and interpreted the data; Wrote the paper.

Liesbeth Jansen: Conceived and designed the experiments; Analyzed and interpreted the data; Contributed reagents, materials, analysis tools or data; Wrote the paper.

Michelle E Hartmans: Performed the experiments; Analyzed and interpreted the data; Wrote the paper.

Geertruida de Bock: Analyzed and interpreted the data; Contributed reagents, materials, analysis tools or data; Wrote the paper.

Paul M N Werker and Joost M Klaase: Contributed reagents, materials, analysis tools or data; Wrote the paper.

### Funding statement

This research did not receive any specific grant from funding agencies in the public, commercial, or not-for-profit sectors.

### Data availability statement

Data will be made available on request.

### Declaration of interests statement

The authors declare the following conflict of interests: Paul Werker is member of a SERB for Fidia Ltd, Milan Italy.

### Additional information

No additional information is available for this paper.
